# Market and welfare effects of a nationwide sugar-sweetened beverage tax in the U.S.

**DOI:** 10.3389/fpubh.2026.1755355

**Published:** 2026-05-15

**Authors:** Yunkyung Lee, Konstantinos Giannakas

**Affiliations:** 1 Agribusiness and Food Industry Management, California State Polytechnic University, Pomona, CA, United States; 2 Agricultural Economics, University of Nebraska-Lincoln, Lincoln, NE, United States

**Keywords:** heterogeneous consumers, heterogeneous producers, market and welfare effects, sin tax, soda tax, sugar-sweetened beverage tax

## Abstract

**Introduction:**

Sugar sweetened beverage (SSB) taxes have been widely proposed and implemented as public health policies to reduce sugar consumption and improve population health. However, the system wide market and welfare impacts of such taxes, particularly when accounting for interactions across related beverage markets and heterogeneous economic agents, remain insufficiently understood. This study aims to evaluate the market and welfare effects of introducing a nationwide SSB tax in the U.S., with particular attention to the distributional impacts on consumers, producers, and related supply chains.

**Methods:**

To analyze the system wide market and welfare impacts of a nationwide SSB tax in the U.S., the paper develops an integrated multi market framework of heterogeneous consumers, heterogeneous producers, and imperfectly competitive beverage companies. Analytical and simulation results are used to evaluate the impacts of the tax.

**Results:**

Analytical and simulation results reveal that the increased prices of soda and fruit juice that follow the tax introduction cause significant losses to consumers (the intended policy beneficiaries) and soda manufacturers, and gains in the fruit juice supply chain. The analysis also shows that, even after accounting for potential health related cost savings from reduced SSB consumption, a nationwide SSB tax in the U.S. will impose welfare losses on consumers.

**Discussion:**

The results indicate that reinvesting SSB tax revenues in public health related programs can not only offset these losses but also generate substantial net welfare gains for consumers. While the analysis is conducted in the U.S. context, the proposed modeling framework is more general and can be readily adapted to assess the incidence and welfare effects of SSB taxes in other regions or countries.

**JEL classification codes:**

**Q18, H0, H22**

## 1 Introduction

The sugar-sweetened beverages (SSB) have been the single largest source of added sugars in the U.S. consumers' diet since the 1970s (1). Despite a steady decline in SSB consumption from 2003 to 2018, the overall SSB intake remains high, particularly among overweight adults and children (2, 3). In particular, the average daily caloric intake of SSB among adults and children is 262.5 kcal, which represents 11.4 percent of an adult's total daily calorie consumption (2, 4). Excessive SSB consumption is associated with increased risk of obesity, diabetes, cardiovascular diseases and dental caries, and has prompted calls for reduced sugary drink consumption to improve population health outcomes (5).

The SSB tax policy is considered one of the key weapons in the quest to discourage unhealthy beverage consumption (6, 7). In the context of SSB tax policies, taxable beverages typically include soda, sports drinks, energy drinks, and sweetened iced teas, while exempt beverages include milk-based drinks, fruit juice and alcohol (8). By decreasing the consumption of SSB, SSB taxes can lower the incidence of diabetes, obesity and cardiovascular diseases, people who are at risk and those already ill can benefit from this policy (9, 10). Reducing the incidence of those diseases can also decrease government expenses for public health treatments, which were about $157 billion in 2018 (11). The potential health benefits and tax revenues have made the SSB tax an increasingly appealing policy response to these diseases in the U.S. (12).

Despite its potential benefits, the effectiveness and desirability of the SSB tax have been a controversial issue in the literature. In particular, even though the policy can reduce the local consumption of SSB, its effectiveness can be reduced by increased cross-border shopping, especially in taxing cities that compete with nearby untaxed suburbs (11, 13–15). In addition, the tax could lead to an increase in the costs of the beverage industry from $0.92 billion to $49.72 billion if the sector absorbed half of the tax burden (16). Evidence from international studies, including Brazil, further suggests that SSB taxation can generate unintended substitution effects, as consumers shift toward untaxed alternatives such as imported SSBs or other high-sugar products, potentially offsetting health gains and even increasing overall calorie intake (17, 18). These concerns have led to growing skepticism toward locally implemented SSB taxes and increased interest in broader regional or national policy approaches.

While a national SSB tax could mitigate cross-border shopping (and its undesirable effect on SSB consumption), its broader economic impacts are unexamined in existing research. Literature to date concentrates on local SSB taxes in various U.S. cities [see Cawley et al. (11)]. This paper aims to bridge the gap by evaluating the economic implications of introducing an SSB tax on a national scale.

In addition to focusing on the economic effects of a nationwide SSB tax in the U.S., a central contribution of this study is the explicit consideration of consumer heterogeneity, together with heterogeneous producers across the SSB supply chain and related markets for non-SSB products such as fruit juice. This way, our study provides a more holistic approach to the analysis of SSB taxes that has been missing from the relevant literature (15). While some studies document heterogeneous consumer responses to SSB taxes by race, income, and preferences (11, 13, 14, 19), and others analyze nationwide SSB taxes using partial or general equilibrium models to assess impacts on calorie intake and industry revenue (17, 20), the distributional effects of the tax across different consumers and producers of SSBs and their substitutes remain largely unexplored.

The key objective of this paper is the determination of the system-wide market and welfare impacts of the introduction of a nationwide SSB tax in the U.S, with a focus on how the tax affects beverage prices, consumer choices, producer decisions, and the welfare/economic wellbeing of consumers and producers. In particular, this study seeks to (1) determine the impact of the SSB tax on the markets for sugary drinks and their substitute products (like 100% fruit juice) and the welfare of the interest groups involved (i.e., consumers, producers, and suppliers of sugary drinks and the substitute products), and (2) quantify the theoretical findings using actual data from the U.S. soda and 100% fruit juice markets, which represent SSB and non-SSB markets, respectively.

To analyze the market and welfare effects of the SSB tax, the study develops an integrated, multi-market framework that explicitly accounts for (a) differences in consumer preferences for sodas and fruit juices, (b) differences in producer agronomic characteristics, and (c) imperfect competition among soda and fruit juice firms. This framework is an adaptation of the Giannakas (21) framework of heterogeneous agents and enables the disaggregation of the welfare impacts of the SSB tax as well as the determination of the cross-market effects of the policy throughout the SSB and non-SSB value chains that, as mentioned earlier, have been ignored by the relevant literature. Once developed, the theoretical framework is (i) calibrated using price, production cost and quantity data, and estimated market power of soda and fruit juice manufacturers between 2015 and 2018, (ii) simulated to quantify the market and welfare impacts of the nationwide SSB tax, and (iii) extended to examine the net change in consumer welfare when the main health benefits from reduced SSB consumption are included in the analysis. It is important to note that, while our analysis focuses on the introduction of a nationwide SSB tax in the U.S., the framework of analysis is more general and can be adapted to study the incidence of SSB taxes in any region/country of interest.

Our analysis shows that the SSB tax increases the consumer and manufacturer prices in both the soda and fruit juice markets, while reducing soda manufacturers' profits by 68% and increasing fruit juice manufacturers' profits by 60%. The higher prices impose economic costs on consumers, with the largest burden falling on soda consumers who continue to purchase soda after the SSB tax, followed by fruit juice consumers who remain loyal to their beverage choice. Even after accounting for potential health-related cost savings from reduced SSB consumption, a nationwide SSB tax in the U.S. will impose welfare losses on consumers. However, our results indicate that reinvesting SSB tax revenues in public health–related programs can not only offset these losses but also generate substantial net welfare gains for consumers.

The rest of the paper is organized as follows. In the next section, the theoretical framework of heterogeneous consumers, heterogeneous producers, and imperfectly competitive firms is developed to analyze their decisions, profits, and welfare before the introduction of the SSB tax. In the section following, we introduce the nationwide SSB tax policy in the soda market and analyze its market and welfare impacts. The simulation analysis is presented before the final section summarizes and concludes the paper.

## 2 Equilibrium conditions before the introduction of the SSB tax

### 2.1 SSB and non-SSB consumers' decisions and welfare

Consumers, in our case, have a choice between three vertically- (or quality-) differentiated products (22): sugar-added beverages (soda), non-sugar-added beverages (100% fruit juice; fruit juice, hereafter), and other beverages (water or milk beverages). Let α ∈ [0, *c*] be the differentiating attribute capturing the heterogeneity in consumer valuation of the different beverages. Assuming consumers spend a small share of their income in purchasing beverages, the consumer utility function can be written as:


$$\begin{array}{l} U_{s}=U-P_{s}^{c}+\text{λ}\alpha \text{ if a unit of soda isconsumed}\\ U_{j}=U-P_{j}^{c}+\mu \alpha \text{ if a unit of fruit juice isconsumed}\\ U_{o}=U\text{ if a unit of other beverages is consumed} \end{array}$$ (1)

where *U*_*s*_, *U*_*j*_, *and U*_*o*_ are the utilities associated with the unit consumption of soda, fruit juice, and other beverages, respectively; *U* is the base level of utility associated with beverage consumption; $P_{s}^{c}$ and $P_{j}^{c}$ are the consumer prices of soda and fruit juice, respectively; and λ and μ are preference parameters associated with the consumption of soda and fruit juice, respectively. The quality difference between soda and fruit juice is captured by the assumption λ < μ with (μ−λ)α capturing the difference in the valuation of fruit juice and soda of the consumer with differentiating attribute α–the greater is α, the stronger the consumer preference for high quality beverages. For simplicity and tractability, we assume that the utility associated with the consumption of other beverages, *U*_*o*_, is equal to the base level of utility.

**Figure 1:**
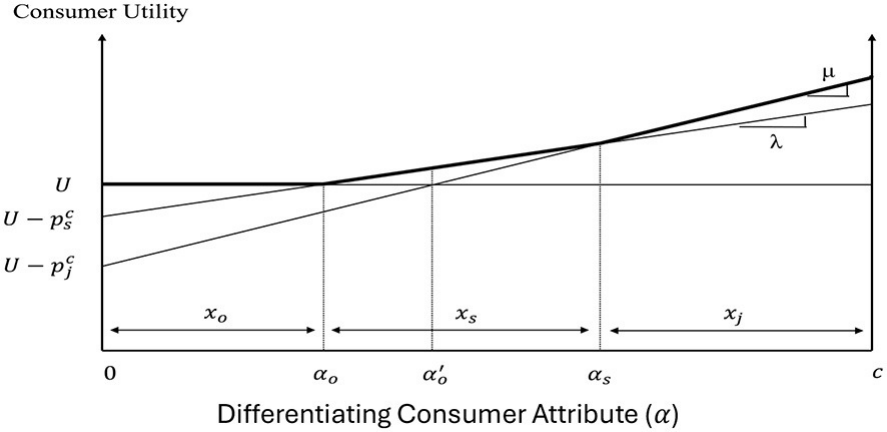
Consumer decisions and welfare.

Figure 1 graphs the utilities associated with the consumption of the different beverages and the consumer purchasing decisions when the three products coexist in the market. Consumers with α ∈ [0, α_*o*_) prefer other beverages, consumers with α ∈ (α_*o*_, α_*s*_) prefer soda, while consumers with α ∈ (α_*s*_, *c*] prefer the fruit juice. When consumers are uniformly distributed between the polar values of α, *c*−α_*s*_ captures the consumer demand for fruit juice, *x*_*j*_, and α_*s*_−α_*o*_ determines the consumer demand for soda, *x*_*s*_, which can be expressed mathematically as in Equation 2:


$$\begin{array}{l} x_{s}=\frac{p_{j}^{c}-p_{s}^{c}}{\mu -\text{λ}}-\frac{p_{s}^{c}}{\text{λ}}=\frac{\text{λ}p_{j}^{c}-\mu p_{s}^{c}}{\text{λ}\left(\mu -\text{λ}\right)}\;\text{and }x_{j}=c-\frac{p_{j}^{c}-p_{s}^{c}}{\mu -\text{λ}}\\ \;=\frac{c\left(\mu -\text{λ}\right)+p_{s}^{c}-p_{j}^{c}}{\mu -\text{λ}} \end{array}$$ (2)

Figure 1 enables us to also derive the welfare of different consumer groups when soda, fruit juice, and other beverages coexist in the market. As Equation 1 captures the utility associated with the consumption of different beverages for the consumer with differentiating attribute α, the area under the effective bold kinked utility curve in Figure 1 captures the welfare of soda consumers ($U_{s}^{*})$, the welfare of fruit juice consumers ($U_{j}^{*}$ and the welfare of other beverage consumers ($U_{o}^{*})$, given by Equations 3–5.


$$\begin{array}{l} U_{s}^{*}=\int_{\alpha_{o}}^{\alpha_{s}}U_{s}d\alpha =Ux_{s}+\frac{1}{2}\text{λ}x_{s}^{2}=\left[U+\frac{\text{λ}p_{j}^{c}-\mu p_{s}^{c}}{2\left(\mu -\text{λ}\right)}\right]\frac{\text{λ}p_{j}^{c}-\mu p_{s}^{c}}{\text{λ}\left(\mu -\text{λ}\right)} \end{array}$$ (3)

$$\begin{array}{l} U_{j}^{*}=\int_{\alpha_{s}}^{c}U_{j}d\alpha =\left(U+\text{λ}x_{s}\right)x_{j}+\frac{1}{2}\mu x_{j}^{2}\\ \;=\left[U+\frac{\begin{array}{c} c\mu \left(\mu -\text{λ}\right)\\ +\left(2\text{λ}-\mu \right)p_{j}^{c}-3\mu p_{s}^{c} \end{array}}{2\left(\mu -\text{λ}\right)}\right]\frac{c\left(\mu -\text{λ}\right)-p_{j}^{c}+p_{s}^{c}}{\mu -\text{λ}} \end{array}$$ (4)

$$\begin{array}{l} U_{o}^{*}=\int_{0}^{\alpha_{o}}U_{o}d\alpha =U\alpha_{o}=\frac{Up_{s}^{c}}{\text{λ}} \end{array}$$ (5)

### 2.2 SSB and non-SSB input producers' decisions and welfare

#### 2.2.1 Crop producers

Crop producers are potential input suppliers in the production of high fructose corn syrup (HFCS) used in soda production. They have three different choices for crop production—corn, soybeans,^1^ and an alternative crop—and they differ in their costs of producing these crops. Letting *A* ∈ [0, *a*] be the producers' differentiating attribute (with *A* = 0 corresponding to the most efficient producer and *A* = *a* to the least efficient one), their net returns function can be expressed as in Equation 6:


$$\begin{array}{l} NR_{b}=P_{b}-w_{b}-\gamma A\text{ if a unit of soybeans is produced}\\ NR_{c}=P_{c}-w_{c}-\delta A\text{ if a unit of corn is produced}\\ NR_{oc}=0\text{ if a unit of an alternative crop is produced} \end{array}$$ (6)

where *P*_*b*_ and *P*_*c*_ are the producer prices of soybeans and corn, respectively; *w*_*b*_ and *w*_*c*_ are the production costs for soybeans and corn, respectively, that are exogenous to producers; and γ and δ are non-negative cost enhancement factors associated with the production of soybeans and corn, respectively. For simplicity, the net returns from the alternative crop are normalized to zero. To capture the higher cost of producing soybeans (24), we assume γ > δ, with (γ−δ)*A* capturing the difference in the idiosyncratic costs of producing soybeans and corn for the producer with differentiating characteristic *A*.

**Figure 2:**
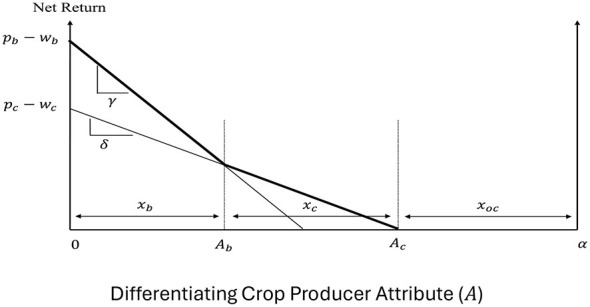
Crop producer decisions and welfare.

Figure 2 graphs the net returns from the different options and the crop producers' decisions when the different crops coexist in the market. More efficient producers with *A* ∈ [0, *A*_*b*_] find it optimal to grow soybeans, producers with *A* ∈ (*A*_*b*_, *A*_*c*_] grow corn, while the least efficient producers with *A* ∈ (*A*_*c*_, *a*] grow the alternative crop. Assuming that producers are uniformly distributed between the polar values of *A*, *A*_*b*_ determines the supply of soybeans, *x*_*b*_, and *A*_*c*_−*A*_*b*_ determines the supply of corn, *x*_*c*_, given by Equation 7 as:


$$\begin{array}{l} x_{b}=\;\frac{\left(p_{b}-w_{b\;}\right)-\;\left({p_{c}-w}_{c\;}\right)}{\gamma -\delta }\;\text{and}\\ x_{c}=\;\frac{\gamma \left(p_{c}-w_{c}\right)-\delta \left(p_{b}-w_{b}\right)}{\delta \left(\gamma -\delta \right)}\; \end{array}$$ (7)

Note that, in addition to depicting farmers' production decisions, Figure 2 can be used to determine the welfare of the different producers. Specifically, the area under the bold kinked net returns curve captures the welfare of soybean producers ($NR_{b}^{*})$ and the welfare of corn producers ($NR_{c}^{*})$, given by Equations 8 and 9 as:


$$\begin{array}{l} NR_{b}*=\int_{0}^{A_{b}}NR_{b}dA=\left(p_{b}-w_{b}-\frac{1}{2}\gamma x_{b}\right)\;x_{b}\\ \;=\frac{\begin{array}{c} \left[\left(\gamma -2\delta \right)\left(p_{b}-w_{b}\right)+\gamma \left(p_{c}-w_{c}\right)\right]\\ \left[\left(p_{b}-w_{b}\right)-\left(p_{c}-w_{c}\right)\right] \end{array}}{2{\left(\gamma -\delta \right)}^{2}} \end{array}$$ (8)

$$\begin{array}{l} NR_{c}*=\int_{A_{b}}^{A_{c}}NR_{c}dA=\frac{1}{2}\left(p_{c}-w_{c}-\delta x_{b}\right)x_{c}\\ \;=\frac{{\left[\gamma \left(p_{c}-w_{c}\right)-\delta (p_{b}-w_{b})\right]}^{2}}{2\delta {\left(\gamma -\delta \right)}^{2}} \end{array}$$ (9)

#### 2.2.2 Fruit producers

Fruit producers can produce fresh fruit, processed fruit, or other types of fruit and their choice depends on the net returns associated with the production of the different kinds of fruit. Processed fruits are used for fruit juice production, whereas other types of fruit are used for canned or dried fruit production. Let *B* ∈ [0, *b*] be the fruit producers' differentiating attribute with *B* = 0 corresponding to the most efficient producer and *B* = *b* to the least efficient one. Normalizing the net returns to the other kinds of fruit to zero, producers' net returns function can be expressed as in Equation 10:


$$\begin{array}{l} NR_{f}=P_{f}-w_{f}-\varepsilon \;B\text{ if a unit of fresh fruit isproduced}\\ NR_{p}=P_{p}-w_{p}-\upsilon \;B\text{ if a unit of processed fruit is produced}\\ NR_{of}=0\text{ if a unit of other types of fruit is produced} \end{array}$$ (10)

where *P*_*f*_ and *P*_*p*_ are the producer prices of fresh and processed fruit, respectively; *w*_*f*_ and *w*_*p*_ are the exogenous production costs, and ε and υ are cost enhancement factors associated with the production of fresh and processed fruit, respectively. We assume that ε>υ with (ε−υ)*B* capturing the difference in the costs of producing fresh and processed fruit for the producer with differentiating attribute *B*.

**Figure 3:**
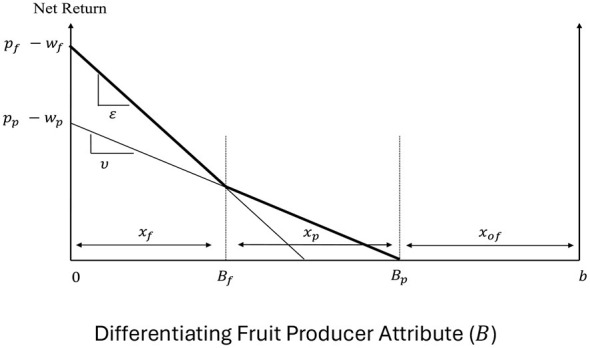
Fruit producer decisions and welfare.

Figure 3 graphs the net returns associated with the different options and the fruit producers' decisions when the different products coexist in the market. More efficient producers with *B* ∈ [0, *B*_*f*_] grow fresh fruit, producers with *B* ∈ (*B*_*f*_, *B*_*p*_] grow processed fruit, while the least efficient producers with *B* ∈ (*B*_*p*_, *b*] grow alternative types of fruit. When producers are uniformly distributed between the polar values of *B*, *x*_*f*_ (= *B*_*f*_) and *x*_*p*_ (= *B*_*p*_−*B*_*f*_) give the supplies of fresh and processed fruit, as given by Equation 11:


$$\begin{array}{l} x_{f}=\;\frac{\left(p_{f}-w_{f\;}\right)-\;\left({p_{p}-w}_{p\;}\right)}{\varepsilon -\upsilon }\;\text{and}\\ x_{p}=\;\frac{\varepsilon \left(p_{p}-w_{p}\right)-\upsilon \left(p_{f}-w_{f}\right)}{\upsilon \left(\varepsilon -\upsilon \right)} \end{array}$$ (11)

The welfare of fruit producers is given by the area under the effective bold kinked net returns curve in Figure 3. Specifically, the welfare of fresh fruit producers ($NR_{p}^{*})$ and the welfare of processed fruit producers ($NR_{f}^{*})$ are given by Equations 12 and 13 as:


$$\begin{array}{l} NR_{f}^{*}=\int_{0}^{B_{f}}NR_{f}dB=\left(p_{f}-w_{f}-\frac{1}{2}\varepsilon x_{f}\right)\;x_{f}\\ \;=\frac{\begin{array}{c} \left[\left(\varepsilon -2\upsilon \right)\left(p_{f}-w_{f}\right)+\varepsilon \left(p_{p}-w_{p}\right)\right]\\ \left[\left(p_{f}-w_{f}\right)-\left(p_{p}-w_{p}\right)\right] \end{array}}{2{\left(\varepsilon -\upsilon \right)}^{2}} \end{array}$$ (12)

$$\begin{array}{l} NR_{p}^{*}=\int_{B_{f}}^{B_{p}}NR_{p}dB=\frac{1}{2}\left(p_{p}-w_{p}-\upsilon x_{f}\right)x_{p}\\ \;=\frac{{\left[\varepsilon \left(p_{p}-w_{p}\right)-\upsilon \left(p_{f}-w_{f}\right)\right]}^{2}}{2\upsilon {\left(\varepsilon -\upsilon \right)}^{2}} \end{array}$$ (13)

### 2.3 SSB and non-SSB firms' decisions

#### 2.3.1 Soda firms

As noted earlier, corn producers supply corn to HFCS processors, and those processors supply corn syrup to soda firms. HFCS is manufactured with *corn* and *other inputs* (e.g., inputs for wet milling process) and is found in about 90% of soda products (25). Corn is processed to corn starch, which is combined with fungi to extract glucose and fructose, which become HFCS. Soda firms combine HFCS with *other inputs* (e.g., carbonated water and coloring ingredients) to produce the final soda products.

**Figure 4:**
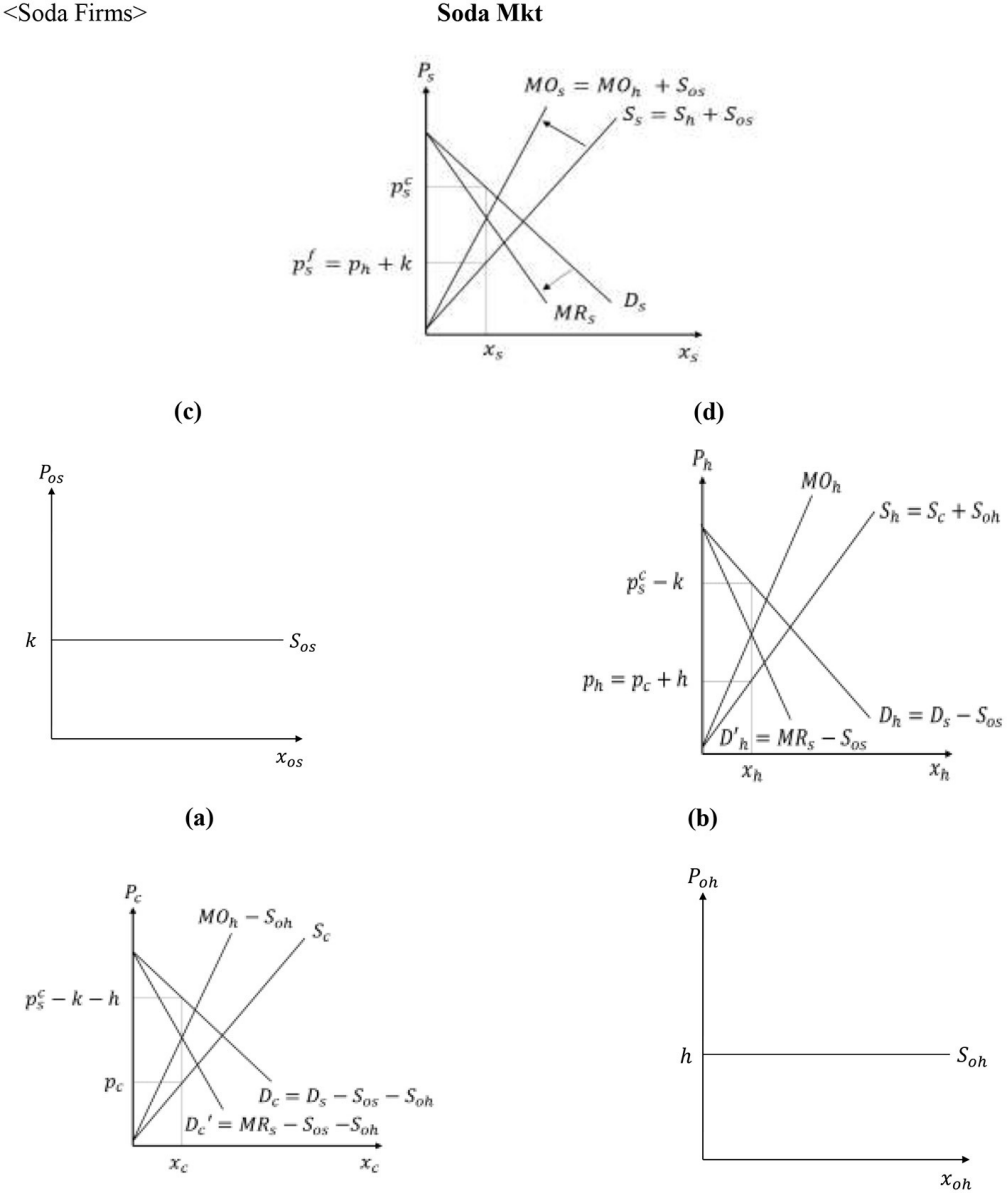
Determination of soda firm, HFCS processor, and corn producer prices. Inputs for HFCS: **(a)** Corn Mkt, **(b)** Other Inputs Mkt. Inputs for soda: **(c)** Other Inputs Mkt, **(d)** HFCS Mkt.

Soda firms have oligopsonistic and oligopolistic market power that is exercised when procuring HFCS and when selling soda to the final consumers, respectively (26–29).^2^ As a result, soda firms maximize their profit by producing at a level determined by the equality of marginal outlays and marginal revenues,^3^ as given by Equations 14 and 15:


$$MO_{h}\;=w_{c}+\frac{\delta }{\gamma }\left(p_{b}-w_{b}\right)+\;h+\frac{\delta (\gamma -\delta )}{\gamma }(1+\theta_{h}^{b})x_{h}$$ (14)

$$\begin{array}{l} MR_{s}=\;\frac{\text{λ}}{\mu }p_{j}^{c}-\frac{\text{λ}}{\mu }\left(\mu -\text{λ}\right)\left(1+\theta_{s}^{s}\right)x_{s} \end{array}$$ (15)

with $\theta_{h}^{b}\in \left[0,1\right]$ and $\theta_{s}^{s}\in \left[0,1\right]$ capturing the soda producers' market power when procuring HFCS and when selling soda to consumers, respectively.^4^ Figure 4 graphs the equilibrium conditions in the soda supply channel.

#### 2.3.2 Fruit juice firms

**Figure 5:**
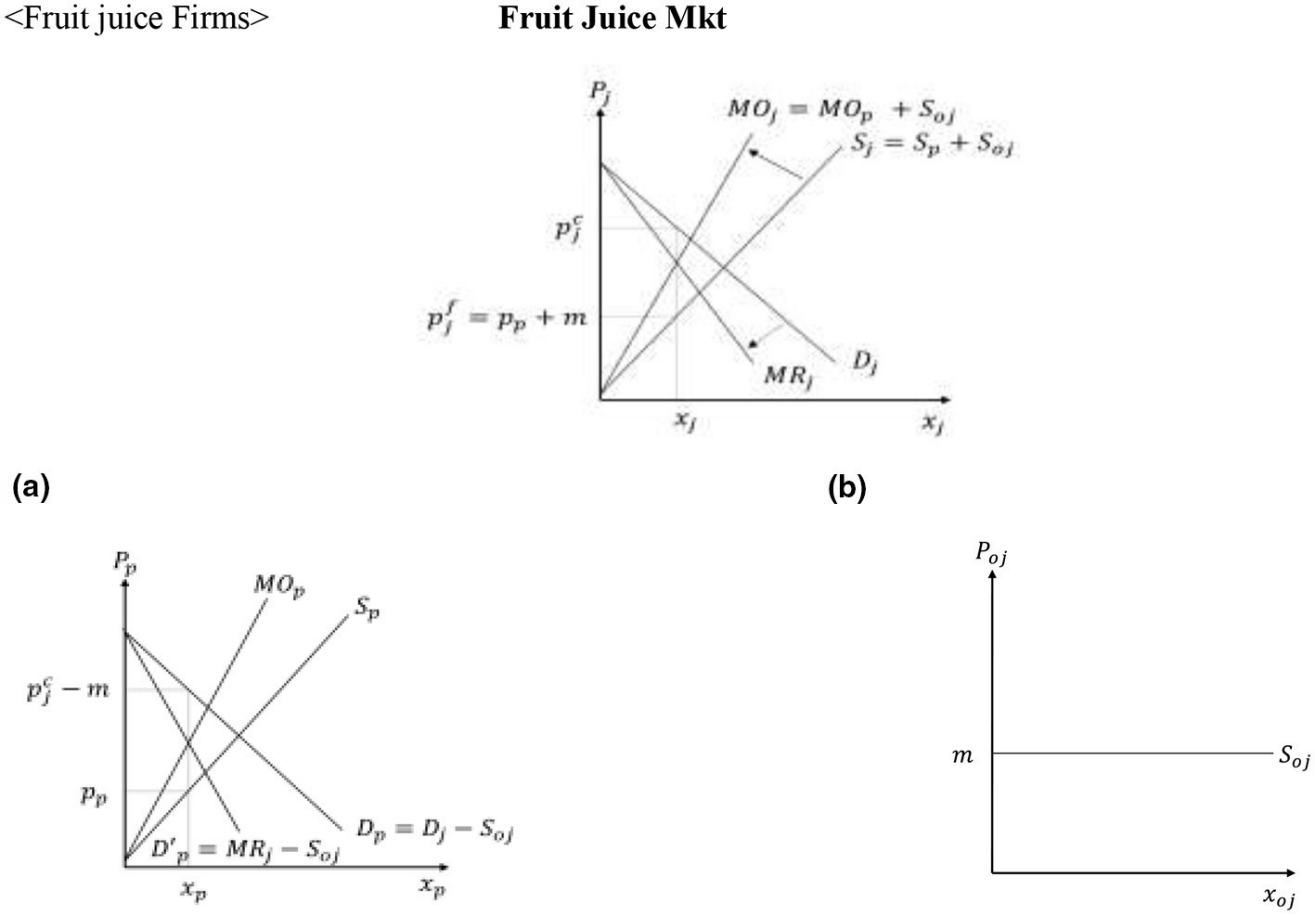
Determination of fruit juice firm and processed fruit producer prices. **(a)** Processed Fruit Mkt. **(b)** Other Inputs Mkt.

Given that fruit juice firms have oligopsonistic and oligopolistic market power, the marginal outlay and marginal revenue curves for fruit juice firms given by Equations 16 and 17:^5^


$$\begin{array}{l} MO_{j}=w_{p}+\frac{\upsilon }{\varepsilon }\left(p_{f}-w_{f}\right)+m+\frac{\upsilon \left(\varepsilon -\upsilon \right)}{\varepsilon }{\left(1+\theta_{p}^{b}\right)x}_{j} \end{array}$$ (16)

$$\begin{array}{l} MR_{j}=c\left(\mu -\text{λ}\right)+p_{s}^{c}-\left(\mu -\text{λ}\right){\left(1+\theta_{j}^{s}\right)x}_{j} \end{array}$$ (17)

where $\theta_{p}^{b}$ and $\theta_{j}^{s}$ capture fruit juice firms' oligopsonistic and oligopolistic market power, respectively. Figure 5 graphs the equilibrium conditions in the fruit juice supply channel, while Appendix 1 in Supplementary material presents the mathematical expressions for the market equilibria in the soda and fruit juice supply chains before the introduction of the SSB tax, depicted in Figures 4, 5.

## 3 Introduction of the SSB tax

**Figure 6:**
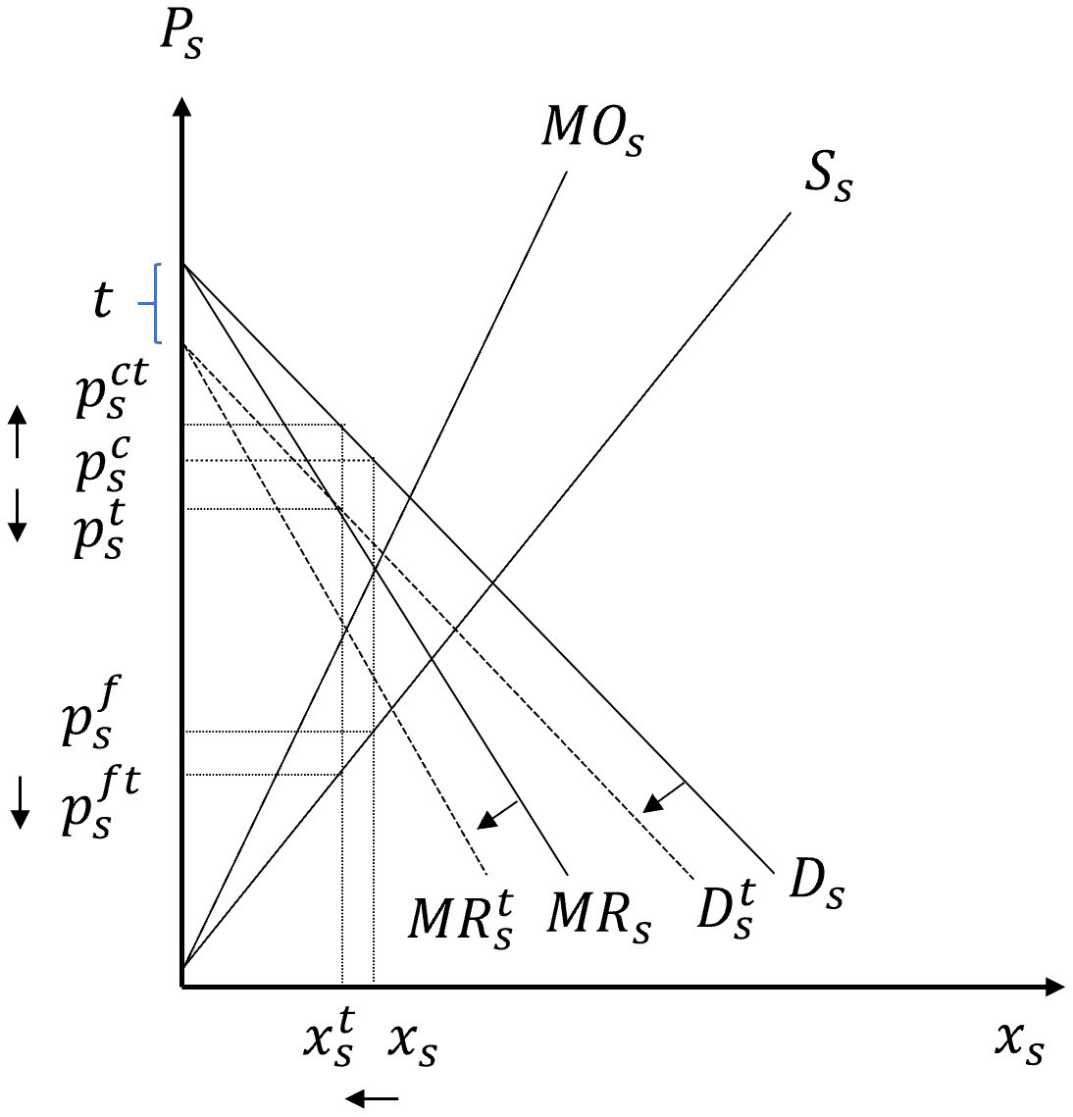
The introduction of the SSB tax in the soda market.

Under the SSB tax, soda beverage distributors are required to pay a unit tax amount of *t*. The unit tax *t* is equal to the price difference between the price paid by soda consumers and the price received by the soda firms. Graphically, the introduction of SSB tax shifts the demand curve *D*_*s*_ to $D_{s}^{t}$, where *D*_*s*_ is the consumer demand for soda (mapping the maximum consumer willingness to pay for soda products) and $D_{s}^{t}$ is the demand curve that soda firms face (which maps the maximum price they can receive for different quantities of their products under the tax). As shown in Figure 6, the introduction of the tax reduces the equilibrium quantity of soda from *x*_*s*_ to $x_{s}^{t}$, increases the consumer price from $p_{s}^{c}$ to $p_{s}^{ct}$, and reduces the price that soda firms receive from $p_{s}^{c}$ to $p_{s}^{t}$ and the costs of these firms from $p_{s}^{f}$ to $p_{s}^{ft}$. Due to these changes, soda consumers and firms lose, while taxpayers benefit from the tax revenues.

### 3.1 System-wide market and welfare effects of the SSB tax

**Figure 7:**
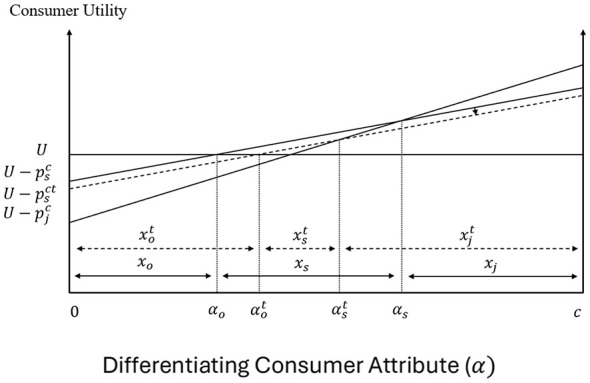
Direct effects of the SSB tax on consumer decisions and welfare.

The direct impact of the SSB tax on consumers stems from the increased price of soda, which reduces the utility associated with the consumption of this product. As Figure 7 shows, an increase in $p_{s}^{c}$ causes a downward parallel shift of *U*_*s*_, which reduces the soda demand from *x*_*s*_ to $x_{s}^{t}$, and increases the demand for fruit juice and other beverages from *x*_*j*_ to $x_{j}^{t}$ and from *x*_*o*_ to $x_{o}^{t}$, respectively. This is because previous soda consumers with α $\in \left(\alpha_{s}^{t},\alpha_{s}\right]$ and α $\in \left(\alpha_{o},\alpha_{o}^{t}\right]$ switch to fruit juice and other beverages, respectively.

**Figure 8:**
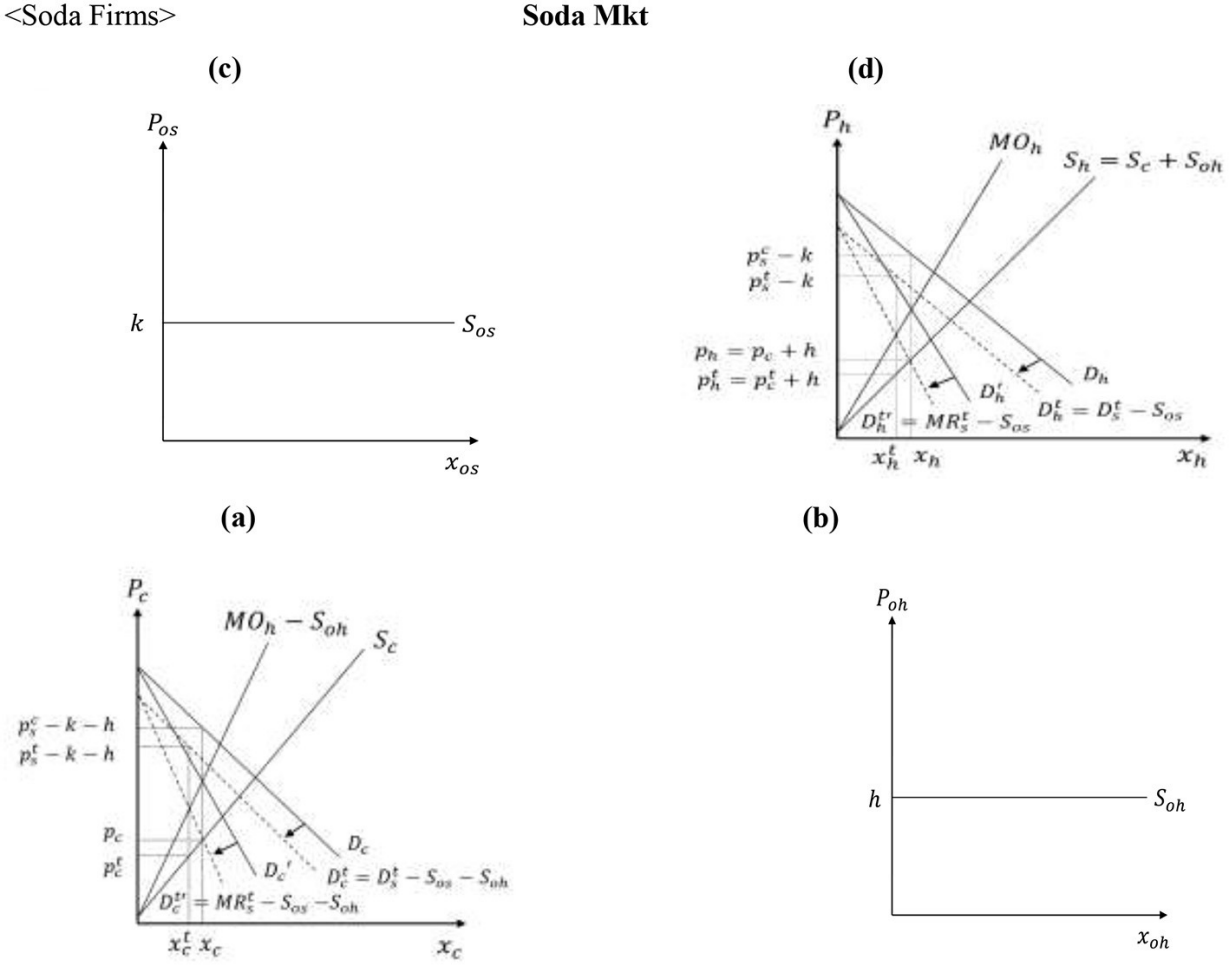
Direct effects of the SSB tax on the soda supply chain. Inputs for HFCS: **(a)** Corn Mkt, **(b)** Other Inputs Mkt. Inputs for soda: **(c)** Other Inputs Mkt, **(d)** HFCS Mkt.

**Figure 9:**
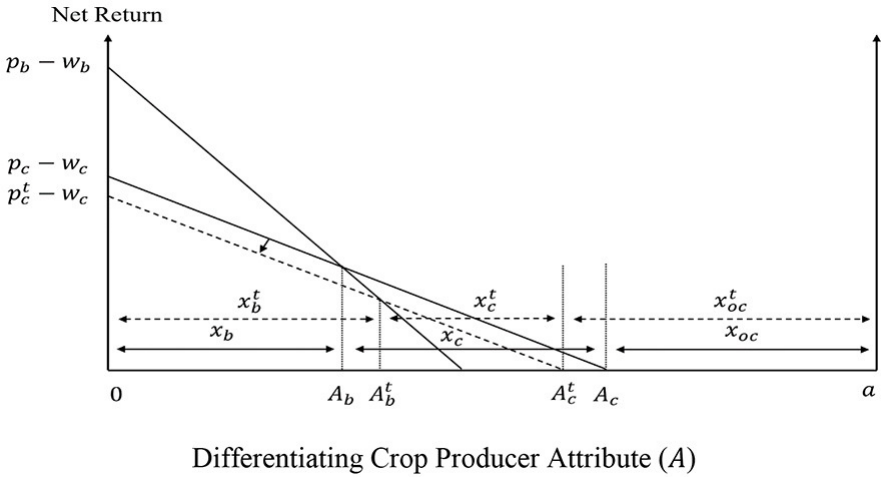
Direct effects of the SSB tax on corn, soybean and other crop producer decisions and welfare.

Figure 8 shows that the downward shift of the demand and marginal revenue curves for soda decreases the price of HFCS from *p*_*h*_ to $p_{h}^{t}$, and reduces the price of corn from *p*_*c*_ to $p_{c}^{t}$, while leaving the prices of other inputs for soda (*k*) and HFCS (*h*) unaffected. The reduced producer price of corn causes a downward parallel shift of the net returns curve associated with corn production and the switching of corn producers with differentiating attributes $A\in \left(A_{b},A_{b}^{t}\right]$ and $A\in \left(A_{c}^{t},A_{c}\right]$ to soybeans and other crops, respectively (see Figure 9).

**Figure 10:**
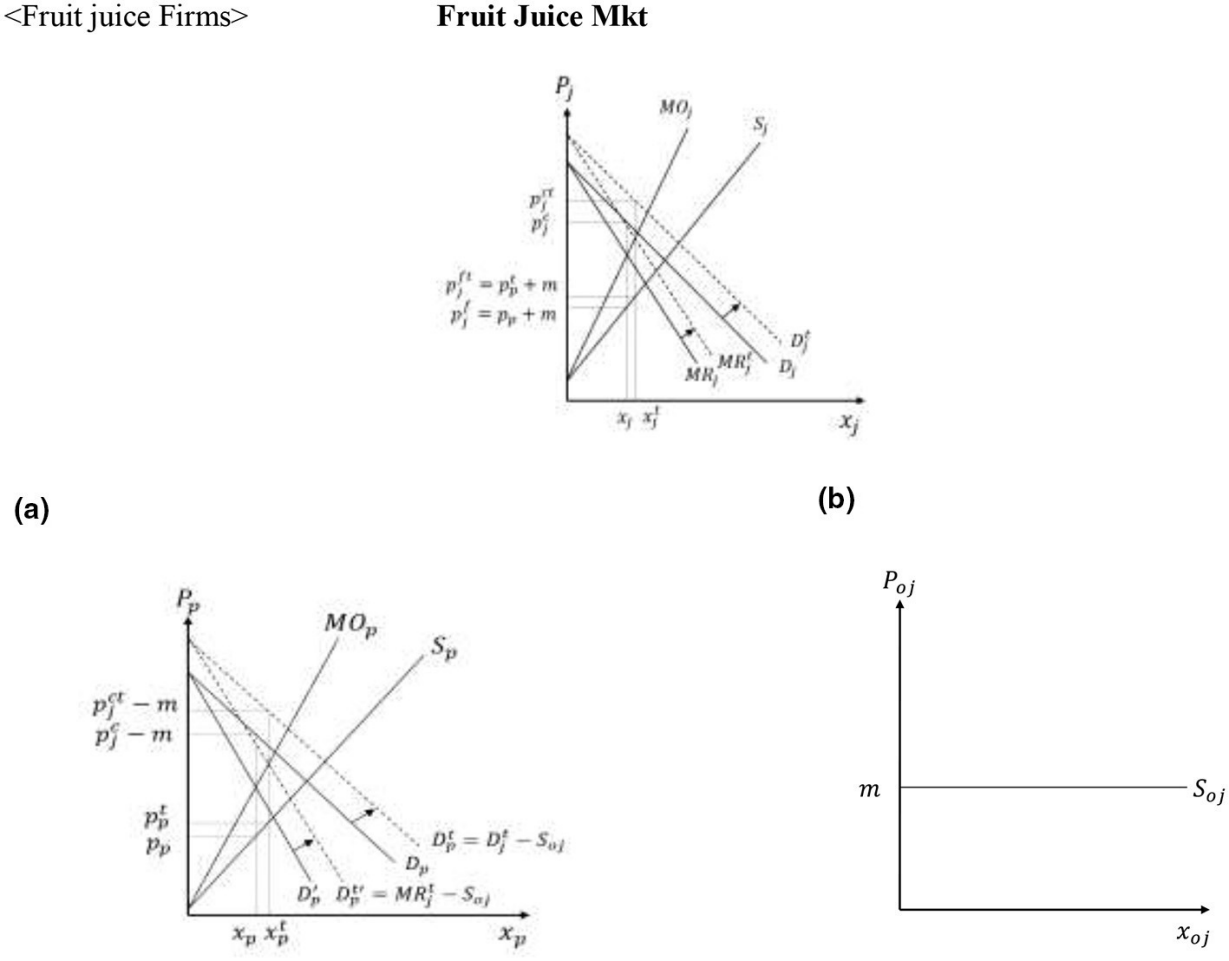
Direct effects of the SSB tax on fruit juice supply chain. **(a**) Processed Fruit Mkt. **(b)** Other Inputs Mkt.

**Figure 11:**
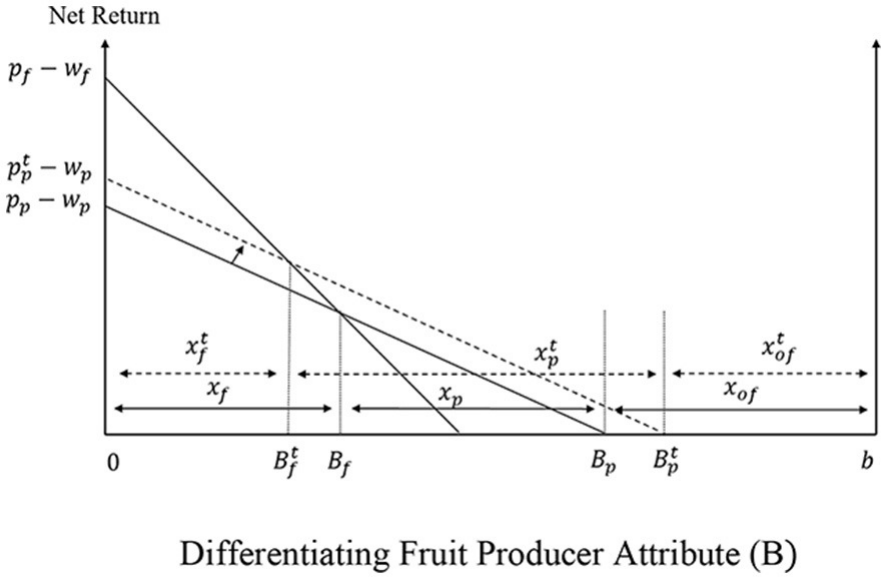
Direct effects of the SSB tax on fruit producer decisions and welfare.

The change in the consumer price of soda in the presence of the tax has also a direct impact on the fruit juice market. In particular, the increased soda price causes soda consumers to switch to alternative beverages and increases the demand for fruit juice. Graphically, the change in fruit juice demand increases the consumer fruit juice price from $p_{j}^{c}$ to $p_{j}^{ct}$, firms' cost from $p_{j}^{f}$ to $p_{j}^{ft}$, and the processed fruit price from *p*_*p*_ to $p_{p}^{t}$ in Figure 10. Figure 11 shows that the increased processed fruit price causes an upward parallel shift of the net returns curve associated with processed fruit production, and the switching of fresh fruit and other types of fruit producers with differentiating attribute $B\in \left(B_{f}^{t},B_{f}\right]$ and $B\in \left(B_{p},B_{p}^{t}\right]$ to the production of processed fruit.

**Figure 12:**
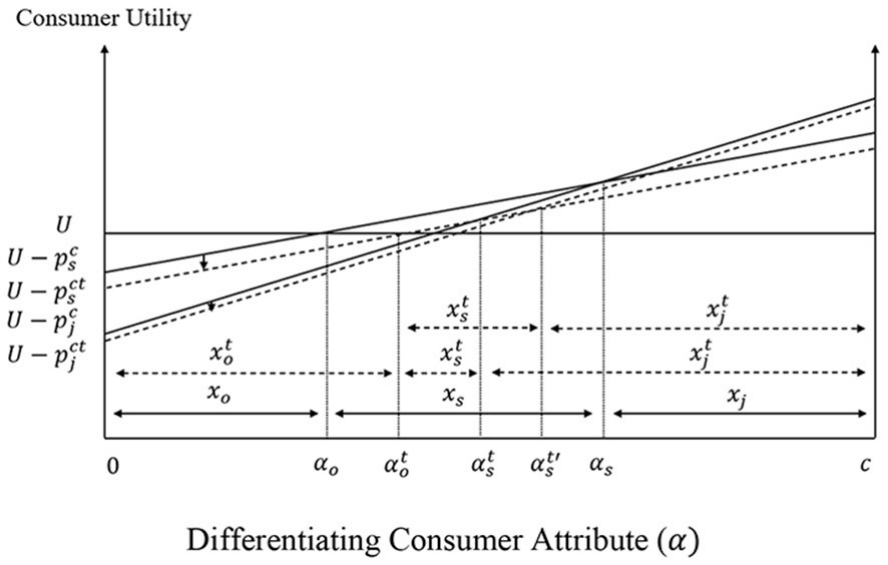
Total effects of the SSB tax on consumer decisions and welfare.

Based on the direct impacts of the SSB tax on consumers, firms and producers, consumers who consume soda before and after the SSB tax lose the most, followed by consumers who switch from soda to other substitutes, and fruit juice consumers. The aggregate consumer loss from the tax is given by Equation 18. (see Figure 12):


$$\begin{array}{l} L_{c}=\int_{\alpha_{o}}^{\alpha_{o}^{t}}\left(U_{s}-U\right)d\alpha +\int_{\alpha_{o}^{t}}^{\alpha_{s}^{t^{\prime }}}\left(U_{s}-U_{s}^{t}\right)d\alpha \\ \;+\int_{\alpha_{s}^{t^{\prime }}}^{\alpha_{s}}\left(U_{s}-U_{j}^{t}\right)d\alpha +\int_{\alpha_{s}}^{c}\left(U_{j}-U_{j}^{t}\right)d\alpha \end{array}$$ (18)

Soda firms lose due to the reduced demand they face under the SSB tax, whereas fruit juice firms gain due to the increased demand for fruit juice. The change in profits of soda and fruit juice firms are given by Equations 19 and 20 as:


$$\begin{array}{l} {\text{Δ}\pi }_{s}=\left(p_{s}^{c}-p_{s}^{t}\right)x_{s}+\left(p_{s}^{t}-p_{s}^{f}\right)\left(x_{s}-x_{s}^{t}\right)-(p_{s}^{f}\\ \;-p_{s}^{ft})x_{s}^{t}<0 \end{array}$$ (19)

$$\begin{array}{l} \text{Δ}\pi_{j}=\left(p_{j}^{ct}-p_{j}^{c}\right)x_{j}^{t}+\left(p_{j}^{c}-p_{j}^{ft}\right)\left(x_{j}^{t}-x_{j}\right)-(p_{j}^{ft}\\ \;-p_{j}^{f})x_{j}>0 \end{array}$$ (20)

Finally, crop producers who produce corn and those who switch from corn to substitutes lose due to the reduced corn prices under the policy, whereas fruit producers who switch from substitutes to processed fruit and those who continue to produce processed fruit gain after the policy is implemented. The magnitude of the loss or gain is determined by the producer differentiating attribute/efficiency in crop or fruit production (see Figures 9, 11). Total crop producers' losses and fruit producers' gains are given by Equations 21 and 22:


$$\begin{array}{l} L_{cp}=\int_{A_{b}}^{A_{b}^{t}}\left(U_{c}-U_{b}\right)dA+\int_{A_{b}^{t}}^{A_{c}^{t}}\left(U_{c}-U_{c}^{t}\right)dA\\ \;+\int_{A_{c}^{t}}^{A_{c}}U_{c}dA<0\; \end{array}$$ (21)

$$\begin{array}{l} G_{p}=\int_{B_{f}^{t}}^{B_{f}}\left(U_{p}^{t}-U_{f}\right)dB+\int_{B_{f}}^{B_{p}}\left(U_{p}^{t}-U_{p}\right)dB\\ \;+\int_{B_{p}}^{B_{p}^{t}}U_{p}^{t}dB>0 \end{array}$$ (22)

Before concluding this section, it is important to note that, in addition to the direct effects of the SSB tax, the changes in prices of soda and fruit juice have feedback effects on the soda and fruit juice supply chains that reduce the magnitude of the impacts of the SSB tax. For example, the tax-induced higher fruit juice prices increase soda demand, which lessens the SSB tax's impact on the soda market. Fruit juice companies and processed fruit producers see higher profits and surplus, respectively, but these benefits are decreased by the resulting feedback effects.

The mathematical expressions for the equilibrium conditions in the soda and fruit juice supply chains in the presence of the SSB tax are derived and reported in Appendix 2 of Supplementary material, and are utilized for our simulation analysis.

## 4 Simulation analysis

### 4.1 Market and welfare effects of the SSB tax policy

The goal of this section is to quantify the system-wide market and welfare effects of the SSB tax policy derived in the previous analysis. We begin by parameterizing the baseline parameters (μ, λ, γ, δ, ε, υ) in the pre-SSB tax market equilibrium conditions using observed data on prices, quantities, market shares, market power and the cost of production for crop and fruit producers. Then, our analysis examines the impact of the SSB tax policy on market equilibrium prices, quantities, firm profits and consumer welfare. We focus on the effects of the introduction of a penny-per-ounce tax on the soda market as this is the rate that most cities have imposed. In particular, the SSB tax has been introduced in nine U.S. cities, with five cities (Berkeley, San Francisco, Oakland and Albany in California; and Cook County in Illinois) having a 1 cent/oz tax, and the other four cities (Boulder, Colorado; Portland, Oregon; Seattle, Washington; and Philadelphia, Pennsylvania) having tax rates ranging between 1.15 and 2 cents/oz.

Table 1 provides the data used for the calibration and simulation of the model. We use consumer price data from Seiler et al. (7) at the UPC/store/week level (a total of 17,582 stores) from January 2015 through September 2018 in Philadelphia, and from Leider and Powell (32) for 581 stores in Cook County, IL, Sacramento and Oakland, CA, collected in late May and June 2017. The pre-tax equilibrium quantities for soda (*x*_*s*_) and fruit juice (*x*_*j*_) are used to reflect the beverage markets that are considered in the theoretical model. We consider the markets for soda (SSB), 100% fruit juice (non-SSB), and bottled water/milk (other types of beverages), while energy drinks, sports beverages, tea, and coffee are not considered in this study. The soybean and corn quantities (*x*_*b*_ and *x*_*c*_), producer prices (*p*_*b*_ and *p*_*c*_), and production costs (*w*_*b*_ and *w*_*c*_) are derived from USDA NASS and ERS for the period 2010–2018. Fresh and processed fruit quantities (*x*_*f*_ and *x*_*p*_), producer prices (*p*_*f*_ and *p*_*p*_) and production costs (*w*_*f*_ and *w*_*p*_) for the period 2014–2018 are collected from various sources. These quantities are derived based on retail weights of total citrus and non-citrus fruits, while producer prices and production costs correspond to apples and oranges only, which represent 70% of the U.S. juice market. Soda firms' cost and fruit juice firms' price ($p_{s}^{f}$ and $p_{j}^{f}$) are derived from the relevant consumer prices and the estimated margins from Yoffie and Wang (33) and Luckstead et al. (34). Yoffie and Wang (33) reported a soda firms' net profit margin of 22.1% in 2004, and we use 27% as the soda firm's margin gradually increased from 10.6% in 2000 to 22.1% in 2004. We utilize the estimated fruit juice firms' margin from Luckstead et al. (34), who analyzed the oligopolistic competition between Florida and São Paulo processors in the U.S. orange juice market. Other parameters such as the price of HFCS (*p*_*h*_) and other inputs costs (*m, k, h*) are calculated using the formulas developed in the crop and fruit producer decision sections. Finally, the economic model includes the market power soda and fruit juice firms are able to exercise on consumers and input suppliers. We use $\theta_{s}^{s}$ = 0.76 (soda firms' market power as seller) and $\theta_{j}^{s}=0.7$ (fruit juice firms' market power as seller) that were estimated by Dhar et al. (27) and Luckstead et al. (34), and supported by other studies on soda manufacturers' market power (35).

**Table 1:** Data description.

Parameter	Value	References
${\text{p}}_{\text{s}}^{\text{c}}$	3.51 cents/oz	(7, 32)
${\text{p}}_{\text{j}}^{\text{c}}$	7.15 cents/oz	(7)
${\text{x}}_{\text{s}}^{\text{a}}$	1,257,108 million oz	(54)
${\text{x}}_{\text{j}}^{\text{b}}$	282,000 million oz	(54, 63)
**p**_**b**_	$10.7/bushel	(64)
**p**_**c**_	$4.29/bushel	(64)
**w**_**b**_	$7.76/bushel	(55, 64)
**w**_**c**_	$2.56/bushel	(55, 64)
${\text{x}}_{\text{b}}^{\text{c}}$	373,735 million oz	(62)
${\text{x}}_{\text{c}}^{\text{c}}$	1,257,108 million oz	(62)
**p**_**f**_	2.35 cents/oz	(63)
**p**_**p**_	0.69 cents/oz	(56, 65)
**w**_**f**_	2.12 cents/oz	(57, 58)
**w**_**p**_	0.55 cents/oz	(57–60)
${\text{x}}_{\text{f}}^{\text{b}}$	495,636 million oz	(61)
${\text{x}}_{\text{p}}^{\text{b}}$	282,000 million oz	(61)
${\text{θ}}_{\text{s}}^{\text{s}}$	0.76	(27)
${\text{θ}}_{\text{j}}^{\text{s}}$	0.70	(34)
${\text{p}}_{\text{s}}^{\text{f}}$	2.55 cents/oz (27% of margin)	(33)
${\text{p}}_{\text{j}}^{\text{f}}$	3.96 cents/oz (45% of margin)	(34)
**p**_**h**_	2.72 cents/oz	
**m**	3.26 cents/oz	
**k**	2.28 cents/oz	
**h**	1.52 cents/oz	
**t**	1 cent/oz	

^a^ Soda, 100% fruit juice, and bottled water and milk account for 36.5%, 8.3%, and 56.2% of the total beverage market, respectively.

^b^ Fresh and processed fruit market share is computed by the retail weight, which is estimated at a primary distribution level, categorized as “fresh” (58%) and “processed with frozen concentrate” (33%). Per capita fruit availability for canned, dried fruits is utilized for calculating the market share of alternative types of fruits. Fruit includes both citrus (oranges, grapefruit, lemons, etc.) and non-citrus (apple, banana, cherries, grapes, papayas, etc.) and the units converted pounds to million oz (1 pound = 16 oz) for the simulation.

^c^ Corn and soybeans account for 37% and 11% of total crop market, respectively, while other type of crops include cotton (46%), wheat (6%), and rice (1%) based on bushels and are converted to million oz (1bushel = 1191.57 oz) for the simulation.

Table 2 provides estimates of the unknown consumer preference parameters (μ and λ) and producer cost enhancement factors associated with the production of soybean (γ), corn (δ), fresh fruit (ε), and processed fruit (υ). We find a small difference between the consumer preference parameters for soda and fruit juice, which is consistent with the U.S. consumers' strong preference for soda compared to other beverages as well as the high market share of soda in the U.S. beverage market. Similarly close are the crop producers' cost enhancement factors, indicating small differences in the idiosyncratic costs (e.g., costs affected by the producer efficiency) of producing corn and soybeans. This is consistent with the fact that soybeans and corn are close substitutes, and producers can share many pieces of equipment in the production of these crops. On the other hand, fruit producers' cost enhancement factors are significantly different due to the requirements of different production practices and significantly greater costs associated with the production of fresh fruit relative to those associated with processed fruit production.

**Table 2:** Calibrated parameters.

Calibrated parameter^a^	Description	Value
μ	Utility enhancement factor (soda)	0.0179
λ	Utility enhancement factor (juice)	0.0105
γ	Cost enhancement factor (soybeans)	0.0036
δ	Cost enhancement factors (corn)	0.0035
ε	Cost enhancement factor (fresh fruit)	10.2
υ	Cost enhancement factor (processed fruit)	0.0226
${\text{θ}}_{\text{h}}^{\text{b}}$	Soda firm's buyer market power	0.25
${\text{θ}}_{\text{p}}^{\text{b}}$	Juice firm's buyer market power	0.35

^a^Parameters are calibrated below 10% variation within the numerical data.

We calibrate soda firms' buyer market power at $\theta_{h}^{b}=0.25$ because the five major HFCS manufacturers (ADM, Cargill, A.E Staley, Cerestar, and CPC) face a relatively elastic corn supply curve (26), while two major buyers utilize most of HFCS for producing sweetened beverages in the market. The fruit juice firms' buyer market power is calibrated at $\theta_{p}^{b}=0.35$. The juice market is also highly concentrated at the processor level (the four-firm concentration ratio was 69% in 2006^6^) and vertically integrated from warehouses to retailers (37). However, their buyer market power is often reduced as processed fruit supply is affected by weather conditions^7^ (38).

Table 3 summarizes the market and welfare effects of the introduction of the nationwide SSB tax in the U.S. The results show that this tax would cause a 23% increase in the consumer soda price, with 76% of the tax passed through to consumers.^8^ Estimates from the literature vary depending on the different tax rates and assumptions utilized. For example, the study of Cook County, IL, found that a penny-per-ounce tax would raise the price of soda by 29% when it is fully (100%) passed through to consumers (39), while the study of Berkeley, CA, found a 47% consumer tax pass-through for the individual size (less than 1L) of SSB (40). The study of Oakland, CA, found a pass-through rate of 82% for bottled soda after a year from the tax implementation, while more recent studies in Philadelphia showed an average tax pass-through rate to consumers of 95% leading to a 34% increase in soda price when a $0.02 per ounce tax rate is imposed (7, 11).

**Table 3:** Market and welfare effects of the SSB tax on the soda and fruit juice supply chains.

SSB tax rate: $0.01/oz	Soda	100% fruit juice
Consumer price ($\Delta \;p_{i}^{ct})$	+23.3%	+11.7%
Price received by firms ($\Delta \;p_{i}^{t}$	−7%	–
Cost of firms ($\Delta \;p_{i}^{ft}$	−0.03%	+19.1%
Equilibrium quantity ($\text{Δ }x_{i}^{t}$)	−61.1%	+69%
Producer price of HFCS (${\text{Δ }p}_{h}^{t}$)	−0.1%	
Producer price of Corn (${\text{Δ }p}_{c}^{t}$)	−0.2%	
Producer price of Processed fruit (${\text{Δ }p}_{p}^{t}$)		+29.5%
Firm profits ($\text{Δ }\pi_{i}^{t}$)	−81.7% (-$5 billion)	+52.9% (+$6.1 billion)
Government annual tax revenue^a^	5.32 billion	
Consumer welfare (*L*_*c*_)	–$8.9 billion (pre-tax market value: $105 billion)	
Consumers of soda	–$4.1 billion	
Consumers of fruit juice	–$0.9 billion	
Consumers of soda switching to other beverages	–$3.9 billion	
Crop producer welfare (*L*_*cp*_)	–$6.8 million (pre-tax market value: $19.7 billion)	
Producers of corn	–$3.8 million	
Producers of corn switching to other crops	–$3 million	
Fruit producer welfare (*G*_*p*_)^b^	+$947.7 million (pre-tax market value: $12.4 billion)	
Producers of processed fruit	+$624.8 million	
Producers of other fruit types to processed fruit	+$322.8 million	

*i* = *s, j* where *s* is soda, *j* is 100% fruit juice + denotes welfare gains, – denotes welfare losses.

^a^This tax revenue is estimated based on the after-tax equilibrium quantity derived from the model.

^b^This welfare change refers to U.S. fruit producers only. As 58.4% of processed fruits (mainly orange and apple) are imported from Brazil, Mexico, and China, further analysis could capture the welfare impacts of the tax on these exporters.

The higher soda price leads to an 11.7% increase in fruit juice price due to a 22.3% reduction in the market share of soda (which represents 61.1% of the initial market share) and a shift of consumer demand to soda substitutes. This results in a reduction in aggregate welfare for beverage consumers of approximately $8.9 billion per year (in 2017 dollars), equivalent to 8.5% of the pre-tax value of the total U.S. beverage market. As shown in the theoretical analysis, the magnitude of the welfare loss is much greater for consumers who continue consuming soda ($4.1 billion or 45% of total consumer welfare loss) and those who switch from soda to other beverages ($3.9 billion or 44% of total consumer welfare loss) under the tax policy, followed by consumers who continue consuming fruit juice beverages ($0.9 billion or 11% of total welfare loss).

Our results are consistent with those of other studies showing a significant increase in soda price and reduced consumption, which result in aggregate consumer welfare loss under the tax policy (32, 41). Unlike these studies, however, our explicit consideration of consumer heterogeneity enables us to identify the disaggregated welfare impacts of the SSB tax on different consumers of SSB and their substitutes.

The profits of soda firms decrease by 81.7% due to the reduced soda firms' price and demand for soda by 23.3% and 61.1%, respectively. With the firms' cost of procuring inputs being affected less by the policy, the significant reduction in soda firms' price decreases the firms' margin and profits. As a result, soda manufacturers experience a substantial contraction in profitability because revenues decline sharply while production costs do not fall proportionally.

A small decline in corn prices (0.2%), resulting from reduced demand for high-fructose corn syrup, reduces aggregate crop producer welfare by approximately $6.8 million, equivalent to 0.03% of the pre-tax value of the total U.S. crop market. This relatively modest impact reflects the fact that the corn market is large and diversified, with multiple end uses such as animal feed, ethanol production, and food processing. Consequently, the reduced demand for corn used in sweetener production represents only a small share of total corn utilization, limiting the overall welfare effect on crop producers.

In addition to increasing the consumer price of juice, the higher demand for fruit juice raises the fruit juice firms' profits by 52.9%. The tax also increases firms' cost by 19.1%, and the processed fruit price by 29.5%. The higher price of processed fruit results in greater economic benefits to producers supplying fruit to juice producers ($1.5 billion or 10.5% of the pre-tax market value of the total fruit market), while there is a relatively small gain for producers who switch from fresh fruit to processed fruit production ($0.3 billion or 2.3% of the pre-tax market value of the total fruit market). The aggregate fruit producers' gain is $1.8 billion that accounts for 12.8% of the total fruit market value prior to the introduction of the tax.

To clarify the effects of soda taxation on soda and fruit juice demand, we compute the own-price elasticity of soda demand and the cross-price elasticity of fruit juice demand with respect to changes in soda prices, using calibrated parameter values. The resulting elasticity estimates are reported in Table 4. As shown in Table 4, the SSB tax substantially reduces soda consumption, with an own-price elasticity of −2.62. This estimate is larger in magnitude than those commonly reported in the empirical literature. For example, a recent meta-analysis of U.S. local SSB taxes reports an average own-price elasticity of −1.5 (42). Evidence from countries outside the U.S. also suggests considerable variation in estimated price responsiveness. For instance, studies from Brazil and Guatemala report own-price elasticities ranging from −1.19 to −3.38 (43, 44).

**Table 4:** Own- and cross-price elasticities with respect to after-tax soda price.

Elasticity of demand	Elasticity value
Own-price elasticity of soda demand	−2.62
Cross-price elasticity of 100% fruit juice demand with respect to soda price	2.96

Elasticities are calculated with respect to changes in the after-tax soda price resulting from the soda tax, using calibrated parameter values from the equilibrium simulation. Percentage changes are measured as equilibrium outcomes, holding all other exogenous factors constant.

Likewise, 100% fruit juice consumption increases significantly in response to higher soda prices, yielding a cross-price elasticity of 2.96. A few studies indicate more narrow substitution effects than those implied by our estimate for the U.S. For example, a study based on a 20% soda tax setting in Mexico reports that a 10% increase in SSB prices leads to an 3.8% increase in fruit juice consumption (45). Similarly, research using demand system estimation in South Africa finds that a 10% increase in the price of soft drinks is associated with a 0.3%−1.4% increase in fruit juice demand, consistent with positive substitution between these categories (46). This empirical range is lower than our simulated estimate, suggesting a stronger substitution response between soda and 100% fruit juice in the U.S. relative to these other countries. The impact of the SSB tax on fruit juice (and size of the relevant cross-price elasticity) is likely also amplified by the fact that our analysis does not explicitly consider other high quality alternatives (like unsweetened tea and coffee), whose consumer appeal is likely captured by the cross-price elasticity of fruit juice, which is the high quality substitute in our study.

### 4.2 Accounting for the health benefits from reduced soda consumption

Even though consumers realize welfare losses from the increased prices under the SSB tax policy, these welfare losses can be offset by the reduced healthcare costs under this policy instrument. This section explores the healthcare cost savings associated with cardiovascular disease, diabetes, BMI and obesity-related diseases from the reduced SSB consumption and their impacts on the welfare analysis of the policy. For the cardiometabolic disease and diabetes outcomes reduction, we use the estimated healthcare cost savings from a $0.01/oz national tax in the U.S. provided by Lee et al. (47), while to account for the healthcare expenditure savings from decreased BMI and obesity-related diseases for children and adults under the same policy, we rely on Long et al. (48).

As our estimate of the impact of a nationwide tax on soda consumption is 11.5% higher than the one used in Long et al. (48)^9^ −22.3% vs. 20%—the annual healthcare cost savings from the decreased BMI and obesity-related diseases for children and adults (that follows the reduced soda consumption due to the nationwide tax) amount, in our case, to $2.63 billion in 2014 dollars (or $3.4 billion in 2023 dollars).^10^ Similarly, as our estimate of the impact of a nationwide tax on soda consumption is 8.25% higher than the one used in Lee et al. (47),^11^ −22.3% vs. 20.6%—the healthcare cost savings from the cardiometabolic disease outcomes reduction and decrease in diabetes amount, in our case, to $1.88 billion of annual savings in 2018 dollars (or $2.28 billion in 2023 dollars).

Based on the simulation analysis of our study, soda consumers who consume soda before and after the SSB tax lose $4.1 billion per year, while consumers who switch from soda to other substitutes lose $3.9 billion per year due to the higher prices that follow the introduction of the SSB tax. Total consumer losses from the market/price effects of the tax are, then, $8 billion in 2017 dollars (or $9.94 billion in 2023 dollars). The results imply that the consideration of the healthcare cost savings from reduced relevant diseases due to the reduced SSB consumption, which are estimated to be $5.68 billion in 2023 dollars,^12^ reduces the annual consumer welfare losses from the policy to $4.26 billion in 2023 dollars.

Beyond private healthcare cost savings associated with improved health outcomes, consumer welfare losses could be further offset if SSB tax revenues were used to finance public health programs/ interventions.^13^ Our simulation results indicate that the SSB tax would generate approximately $5.32 billion in 2017 dollars ($6.61 billion in 2023 dollars) in annual government tax revenue (Table 3), which could help mitigate the annual consumer welfare losses.

Evidence from the literature on the return on investment (ROI) of public health interventions (66–68) indicates that public health spending often yields returns that exceed the initial expenditures in the U.S. and other high-income countries. A systematic review of ROI studies in high-income settings reports that national public health interventions have a median ROI of 27.2, indicating that benefits frequently exceed costs by an order of magnitude (67). At a subnational level, Brown (68) estimates that each $1 invested in California county public health departments yields $67.07–$88.21 in benefits. More recent methodological work also cautions that ROI estimates vary widely depending on perspective, time horizon, and which benefits are monetized—indicating that ROI should be treated as a scenario parameter rather than a single “true” value.

Motivated by this evidence, a sensitivity analysis is conducted to evaluate how consumer welfare outcomes vary under alternative ROI assumptions. For simplicity, we assume that the government revenue from the SSB tax ($6.61 billion in 2023 dollars) is fully reinvested into health care programs that generate consumer welfare benefits. For an ROI/benefit-cost ratio of 1:1, investment of the $6.61 billion of SSB tax revenues into health care programs would result in a $2.35 billion (in 2023 dollars) annual consumer welfare gains from the policy ($6.61 billion in health benefits from the investment of the SSB tax revenues in public health programs minus $4.26 billion in the consumer welfare losses incurred in the absence of this reinvestment, in 2023 dollars). Obviously, the consumer welfare gains increase substantially under the higher ROI scenarios reported in the literature. Specifically, under the ROI of 1:27.2 reported by Masters et al. (67), the consumer welfare gain increases to approximately $175.5 billion (in 2023 dollars), while under the ROI of 1:88.21 reported by Brown (68), the consumer welfare gain increases to approximately $578.8 billion (in 2023 dollars). These findings highlight the sensitivity of welfare outcomes to assumptions regarding the effectiveness of reinvesting tax revenues into public health-related programs.

## 5 Summary and concluding remarks

This paper develops an integrated, multi-market framework of analysis to examine the market and welfare impacts of the introduction of a nationwide SSB tax in the U.S. The framework explicitly accounts for the empirically relevant heterogeneity in consumer preferences for different beverages and agricultural producer agronomic characteristics, and imperfect competition among beverage firms. Analytical and simulation results confirm that taxing soda is an effective way to stimulate consumers' choice of healthier beverage options and reduced soda consumption. This effectiveness of the SSB tax is similar to that of tobacco taxation, which has been proven effective in discouraging tobacco consumption (51).

The analysis also reveals that the increased prices of soda and substitute products that follow the introduction of the tax cause significant losses to soda manufactures and gains for fruit juice companies. Fruit juice firms' profits increase due to the greater demand for high-quality beverages, resulting in welfare gains for fruit producers, while consumers and crop producers realize welfare losses under the tax policy. Beverage consumers, the intended beneficiaries of the policy, lose from the market effects of the policy (i.e., increased beverage prices) the most. While the inclusion of the main healthcare cost savings associated with reduced SSB consumption reduces the consumer welfare losses from the market effects of the tax, the analysis suggests that the consumer welfare losses from the policy remain significant. The analysis also shows that for consumers to benefit from the SSB tax policy, at least some of the tax revenues should be reinvested in public health programs. Such programs have been shown to have significant returns on investment, which could result in consumer welfare gains from the policy.

The results of our study have important implications for policy design and the political economy of SSB taxes. In particular, by providing an understanding of the system-wide market and welfare effects of a nationwide SSB tax, our study can assist policy makers in the design of policy mechanisms that induce the desired consumer behavior. For instance, given the importance of prices in low-income consumers' food and beverage purchases (52), enhancing the affordability of healthy beverage options could help improve the outcomes of the policy. Our study can also inform the positions of the different consumer and producer groups involved toward the provisions of the policy. In general, potential winners and losers from the SSB taxes should be expected to politically position themselves in order to serve their interests. Our analysis can be utilized to shape the position of the different groups toward a nationwide SSB tax policy and provide insights on the political economy of policies governing SSB.

This research is particularly timely given the renewed national focus on improving public health and addressing diet-related diseases under the “Make America Healthy Again (MAHA)” initiative. As policymakers consider fiscal tools to promote healthier consumption patterns, understanding the broader market and welfare implications of SSB taxation becomes increasingly important (53). Our findings offer evidence-based insights that can inform the design of more effective and equitable health policies, ensuring that such interventions advance public health goals without imposing disproportionate welfare costs on consumers.

Our study can be extended in a number of ways. The theoretical consumer model can be expanded to consider different types of substitutes of soda, such as diet soda, coffee, unsweetened tea or sports drinks, which will enable the determination of the market and welfare impacts of the policy in these markets. In addition, the analysis can be extended to account for the involvement of soda manufacturers in multiple markets, including those of soda substitutes that, as shown in our analysis, benefit from the SSB tax. Believing that our framework of analysis can serve as the basis for such studies, we leave these questions and extensions open to future research.

Finally, it is important to reiterate that, while our analysis focuses on the introduction of a nationwide SSB tax in the U.S., the framework of analysis is more general and can be used to determine the market and disaggregated welfare impacts of SSB taxes in any region/country of interest.

## Data Availability

The original contributions presented in the study are included in the article/Supplementary material, further inquiries can be directed to the corresponding author.

## Footnotes

1 Corn and soybeans are typically substitutes for crop producers, with planting decisions based on the relative prices and the quality of land (23).

2 The soft drink manufacturing industry has been dominated by the Coca-Cola Company, PepsiCo, Inc., and the Dr Pepper Snapple Group, which accounted for 64% of the market in 2010 (30).

3 Soda firms charge the maximum price consumers are willing to pay for this quantity, $p_{s}^{c}$, while incurring a cost of $p_{s}^{f}$, which is the summation of the prices of HFCS and other inputs, i.e., $p_{s}^{f}=p_{h}+k$. HFCS price is determined by the prices of corn and other inputs used in its production, i.e., *p*_*h*_ = *p*_*c*_+*h*. Under a fixed proportions technology, the equilibrium quantities of soda, HFCS, and the other inputs are normalized to be equal, i.e., *x*_*s*_ = *x*_*h*_ = *x*_*os*_ = *x*_*c*_ = *x*_*oh*_ where *x*_*h*_ and *x*_*os*_ are the equilibrium quantities of HFCS and other inputs used in soda manufacturing, respectively, and *x*_*oh*_ is the equilibrium quantity of other inputs (*oh*) used in HFCS production. The supply of soda is, then, given by $p_{s}^{f}=p_{h}+k=w_{c}+\frac{\delta }{\gamma }\left(p_{b}-w_{b}\right)+h+k+\frac{\delta \left(\gamma -\delta \right)}{\gamma }x_{s}$.

4 The market power parameters $\theta_{h}^{b}$ and $\theta_{s}^{s}$ are also referred to as conjectural variation elasticities (31). The greater is the value of θ, the greater is the market power of the relevant firms in the relevant markets.

5 Assuming that fruit juice beverages are produced with *processed fruit* and *other inputs* (e.g., packaging), the relationship between the production cost of fruit juice and the prices of the inputs used in its production is given by $p_{j}^{f}=p_{p}+m$, where $p_{j}^{f}$ is the cost of fruit juice firms to produce the final juice product, and *m* is the cost of other inputs. The supply for fruit juice is, then, given as $p_{j}^{f}=p_{p}+m=w_{p}+\frac{\upsilon }{\varepsilon }\left(p_{f}-w_{f}\right)+m+\frac{\upsilon \left(\varepsilon -\;\upsilon \right)}{\varepsilon }x_{j}$.

6 Guci and Brown (36) report that the four-firm concentration ratio increased from 42.6% in 1995 to 68.5% in 2006, and the eight-firm concentration ratio increased from 63% to 96.8% during the same period.

7 The citrus production can be decreased by up to 90% with few days of freezing (38).

8 Sensitivity analysis shows that the pass-through rate varies between 71% to 100% depending on the market power of the firms. When the beverage firm is a monemporist (i.e., monopoly and monopsony), the tax pass-through rate is 100%, while absence of market power results in a 71% of tax pass through rate.

9 Long et al. (48) simulate the impact of the SSB tax on BMI reduction and calculate obesity-oriented healthcare costs by using the Medical Expenditure Panel Survey from Gortmaker et al. (49). This study utilizes per capita consumption (Kcal) data as baseline market share for SSB, milk and 100% juice, and find that a penny-per-ounce SSB tax would cause a 20% reduction of sugary drink consumption, which amounts to $23.61 billion in 2014 dollars in 10-year healthcare cost savings for consumers. This result translates into savings of $2.36 billion annually.

10 An alternative derivation of the same result is as follows. As Long et al. (48) include soda, fruit drinks, sport drinks, low-calorie drinks, nonalcoholic wines/malt and other beverages that contain added caloric sweeteners as SSB, the market share of SSB in their study is 56.8% of which 24.4% is the market share of soda. As a result, the healthcare cost savings attributed to a 20% reduction in soda consumption are $10.14 billion of lifetime savings ($23.61 billion ∗56.8 = $10.14 billion) and $1.01 billion of annual savings ($2.36 billion ∗56.8 = $1.01 billion). As our estimate of the impact of a nationwide tax on the market share of soda is 2.59 times greater than the one used in Long et al. (48) – the 20% reduction in soda consumption reduces the market share of soda by 8.59% in Long et al. (48), while the reduction in the market share of soda in our study is 22.3% – the healthcare cost savings from the decreased BMI for children and adults amount to $2.63 billion in 2014 dollars.

11 Lee et al. (47) use a microsimulation model and data from the National Health and Nutrition Examination Survey (NHANES) for the period 2009–2014 to examine how consumers and the government can save on healthcare costs because of the reduced number of events in chronic disease, acute disease, procedures, stroke, strain-associated adverse events, Type 2 diabetes, and increased life expectancy that follows reduced SSB consumption. Using population data of U.S. adults aged 35–80 years, this study uses different models and estimates a life expectancy of 82.5 and 81.7 years in the absence of the policy. The study finds a lifetime quality-adjusted value of health cost savings for consumers of $143.51 billion in 2018 dollars in their lifetime, which, assuming an average survival of 82.1 years, translates into annual savings of $1.74 billion in 2018 dollars ($\frac{\$143.51\;billion}{82.1\;\left(Years\;of\;survival\;on\;average\right)}$ = $1.74 billion).

12 As noted by a reviewer, the healthcare cost savings considered in our study may represent only a partial estimate of the potential healthcare cost savings from a nationwide SSB tax, as other health impacts of SSB consumption (and the corresponding benefits) are not included in our analysis (e.g., cancer burdens and dental caries). However, given that addressing the primary health impacts of SSB consumption results in aggregate annual benefits of $5.68 billion, it appears unlikely that incorporating these additional, less prominent health effects would generate savings exceeding the $4.26 billion required to offset the negative welfare impact of the SSB tax on consumers and result in net consumer welfare gains from the policy. For example, Du et al. (50) estimate the healthcare costs savings from reductions in cancer mortality following the implementation of an SSB tax to $0.34 billion.

13 We owe this line of thought to a reviewer.
