# Supplementary material for: Market and welfare effects of a nationwide sugar-sweetened beverage tax in the U.S
Source: Front Public Health. 2026 May 15;14:1755355. doi: 10.3389/fpubh.2026.1755355 (PMC13219029; doi:10.3389/fpubh.2026.1755355)
Supplement: Supplementary file 1 [file Data_Sheet_1.pdf]

Appendix 1

Market equilibrium: pre-SSB tax

The equilibrium quantity of soda is determined by the equality $MO_s = MR_s$ and is given by

$$x_s^e = \frac{\gamma\lambda P_j^c - \gamma\mu(w_c + h + k) - \mu\delta(p_b - w_b)}{\delta\mu(\gamma-\delta)\left(1+\theta_h^b\right) + \lambda\gamma(\mu-\lambda)(1+\theta_s^s)} \quad (1)$$

Based on equation (1), the equilibrium quantity of soda depends positively on the consumer price of fruit juice (P_j^c) and the production cost of soybeans (w_b), and negatively on the marginal costs of other inputs (*h* and *k*), the production cost of corn (w_c), the cost enhancement factors (δ and μ), the price of soybeans (p_b), and the market power of soda firms in procuring HFCS and selling soda (θ_h^b and θ_s^s).

Similarly, the equilibrium quantity of fruit juice is determined by the equality $MO_j = MR_j$ and is given by

$$x_j^e = \frac{\varepsilon\left[c(\mu-\lambda) + P_s^c - w_p - m\right] - \upsilon(p_f - w_f)}{\upsilon(\varepsilon-\upsilon)\left(1+\theta_p^b\right) + \varepsilon(\mu-\lambda)\left(1+\theta_j^s\right)} \quad (2)$$

This quantity depends positively on the consumer price of soda (P_s^c) and the production cost of fresh fruit (w_f), and negatively on the production cost of processed fruit (w_p), the marginal cost of other inputs (*m*), the price of fresh fruit (p_f), and the market power of fruit juice firms in buying processed fruit and selling fruit juice products (θ_p^b and θ_j^s).

The equilibrium consumer price of soda is then

$$p_s^{ce} = \frac{\lambda}{\mu} p_j^c - \frac{\lambda}{\mu}(\mu - \lambda)\left[\frac{\gamma\lambda P_j^c - \gamma\mu(w_c + h + k) - \mu\delta(p_b - w_b)}{\delta\mu(\gamma-\delta)\left(1+\theta_s^b\right) + \lambda\gamma(\mu-\lambda)\left(1+\theta_s^s\right)}\right] \quad (3)$$

and it depends positively on the producer price of soybeans, the production cost of corn, and the market power of soda firms, and negatively on the soybean production cost and the marginal costs of other inputs.

The equilibrium consumer price of fruit juice is given by

$$p_j^{ce} = c(\mu-\lambda) + p_s^c - (\mu-\lambda)\left\{\frac{\varepsilon[c(\mu-\lambda)+P_s^c-w_p-m]-\upsilon\left(p_f-w_f\right)}{\upsilon(\varepsilon-\upsilon)\left(1+\theta_p^b\right)+\varepsilon(\mu-\lambda)\left(1+\theta_j^s\right)}\right\} \tag{4}$$

and it depends positively on the cost of processed fruit production, the marginal cost of other inputs, the price of processed fruit and the market power of fruit juice firms, and negatively on the cost of fresh fruit production.

The equilibrium cost of producing soda products represents the firms' expenses for buying other inputs and HFCS (p_h), where the price of HFCS is given by the summation of the price of corn (p_c) and other inputs cost, i.e.,

$$p_s^{fe} = w_c + \frac{\delta}{\gamma}(p_b - w_b) + h + k + \frac{\delta(\gamma-\delta)}{\gamma}\left[\frac{\gamma\lambda P_j^c - \gamma\mu(w_c+h+k) - \mu\delta(p_b-w_b)}{\delta\mu(\gamma-\delta)(1+\theta_s^b)+\lambda\gamma(\mu-\lambda)(1+\theta_s^s)}\right] \tag{5}$$

The equilibrium cost of soda products depends positively on the consumer price of fruit juice[1], and negatively on the market power of soda firms.

The equilibrium price of the fruit juice firms is

$$p_j^{fe} = w_p + \frac{\upsilon}{\varepsilon}\left(p_f - w_f\right) + m + \frac{\upsilon(\varepsilon-\upsilon)}{\varepsilon}\left\{\frac{\varepsilon[c(\mu-\lambda)+P_s^c-w_p-m]-\upsilon\left(p_f-w_f\right)}{\upsilon(\varepsilon-\upsilon)\left(1+\theta_p^b\right)+\varepsilon(\mu-\lambda)\left(1+\theta_j^s\right)}\right\} \tag{6}$$

which expresses the amount firms need to pay for procuring processed fruit and other inputs, and it depends positively on the consumer price of soda, and negatively on the market power of

[1] The greater consumer price of juice increases the demand for, and equilibrium quantity of soda, and it also raises the equilibrium cost for soda firms.

fruit juice firms.

The equilibrium processor price of HFCS (p_h^e), the equilibrium producer price of corn (p_c^e), and the equilibrium producer price of processed fruit (p_p^e) are given by

$$p_h^e = p_s^{fe} - k = w_c + \frac{\delta}{\gamma}(p_b - w_b) + h + \frac{\delta(\gamma-\delta)}{\gamma}\left[\frac{\gamma\lambda P_j^c - \gamma\mu(w_c+h+k) - \mu\delta(p_b - w_b)}{\delta\mu(\gamma-\delta)(1+\theta_s^b) + \lambda\gamma(\mu-\lambda)(1+\theta_s^s)}\right] \quad (7)$$

$$p_c^e = p_s^{fe} - k - h = w_c + \frac{\delta}{\gamma}(p_b - w_b) + \frac{\delta(\gamma-\delta)}{\gamma}\left[\frac{\gamma\lambda P_j^c - \gamma\mu(w_c+h+k) - \mu\delta(p_b - w_b)}{\delta\mu(\gamma-\delta)(1+\theta_s^b) + \lambda\gamma(\mu-\lambda)(1+\theta_s^s)}\right] \quad (8)$$

$$p_p^e = p_j^{fe} - m = w_p + \frac{v}{\varepsilon}(p_f - w_f) + \frac{v(\varepsilon-v)}{\varepsilon}\left\{\frac{\varepsilon[c(\mu-\lambda) + P_s^c - w_p - m] - v(p_f - w_f)}{v(\varepsilon-v)(1+\theta_p^b) + \varepsilon(\mu-\lambda)(1+\theta_j^s)}\right\} \quad (9)$$

Based on the equilibrium consumer prices and firms' costs, the soda and fruit juice firms' profits, π_s and π_j, respectively, are determined by

$$\pi_s = (p_s^{ce} - p_s^{fe})x_s^e$$
$$= \left[\frac{\lambda}{\mu}p_j^c - w_c - \frac{\delta}{\gamma}(p_b - w_b) - h - k\right]\left[\frac{\gamma\lambda P_j^c - \gamma\mu(w_c+h+k) - \mu\delta(p_b - w_b)}{\delta\mu(\gamma-\delta)(1+\theta_s^b) + \lambda\gamma(\mu-\lambda)(1+\theta_s^s)}\right] \quad (10)$$
$$+ \left[\frac{\lambda}{\mu}(\lambda-\mu) - \frac{\delta(\gamma-\delta)}{\gamma}\right]\left[\frac{\gamma\lambda P_j^c - \gamma\mu(w_c+h+k) - \mu\delta(p_b - w_b)}{\delta\mu(\gamma-\delta)(1+\theta_s^b) + \lambda\gamma(\mu-\lambda)(1+\theta_s^s)}\right]^2$$

$$\pi_j = (p_j^{ce} - p_j^{fe})x_j^e$$
$$= \left[c(\mu-\lambda) + p_s^c - w_p - \frac{v}{\varepsilon}(p_f - w_f) - m\right]\left\{\frac{\varepsilon[c(\mu-\lambda) + P_s^c - w_p - m] - v(p_f - w_f)}{v(\varepsilon-v)(1+\theta_p^b) + \varepsilon(\mu-\lambda)(1+\theta_j^s)}\right\} \quad (11)$$
$$+ \left[(\lambda-\mu) - \frac{v(\varepsilon-v)}{\varepsilon}\right]\left\{\frac{\varepsilon[c(\mu-\lambda) + P_s^c - w_p - m] - v(p_f - w_f)}{v(\varepsilon-v)(1+\theta_p^b) + \varepsilon(\mu-\lambda)(1+\theta_j^s)}\right\}^2$$

Replication note: Replication of the pre-tax market equilibrium proceeds as follows: (i) solve equations (1) and (2) to obtain the equilibrium quantities of soda and fruit juice; (ii) substitute these equilibrium quantities into equations (3)–(9) to derive equilibrium consumer prices, firms'

costs, and upstream producer prices; and (iii) compute firms' profits using equations (10) and (11). All calculations are deterministic and can be implemented using standard spreadsheet or mathematical software. To simulate the pre-tax equilibrium outcomes, the parameter values reported in Table 1 can be used.
