# Supplementary material for: Market and welfare effects of a nationwide sugar-sweetened beverage tax in the U.S
Source: Front Public Health. 2026 May 15;14:1755355. doi: 10.3389/fpubh.2026.1755355 (PMC13219029; doi:10.3389/fpubh.2026.1755355)
Supplement: Supplementary file 2 [file Data_Sheet_2.pdf]

Appendix 2

Market equilibrium: post-SSB tax

The equilibrium conditions in the soda supply chain in the presence of the SSB tax are derived as follows. After the SSB tax is imposed in the soda market, the demand and marginal revenue curves shift downward by the amount of tax *t*. Using equations (2, consumer demand for soda (x_s)) and (15) in the main part of the manuscript, soda firms' demand and marginal revenue curves after the tax are

$$D_s^t: \ \ p_s^c = \frac{\lambda}{\mu} p_j^c - t - \frac{\lambda}{\mu}(\mu - \lambda)x_s \tag{1}$$

$$MR_s^t = \frac{\lambda}{\mu} p_j^c - t - \frac{\lambda}{\mu}(\mu - \lambda)(1 + \theta_s^s)x_s \tag{2}$$

Based on the optimality condition $MO_s = MR_s^t$, the equilibrium quantity of soda after the introduction of SSB tax is

$$x_s^t = \frac{\gamma\lambda P_j^{ct} - \mu\gamma t - \gamma\mu(w_c + h + k) - \mu\delta(p_b - w_b)}{\delta\mu(\gamma - \delta)\left(1 + \theta_h^b\right) + \lambda\gamma(\mu - \lambda)(1 + \theta_s^s)} \tag{3}$$

We can derive the post-tax equilibrium consumer price of soda and the price received by the soda firms as

$$p_s^{ct} = \frac{\lambda}{\mu} p_j^{ct} - \frac{\lambda}{\mu}(\mu - \lambda)\left[\frac{\gamma\lambda P_j^{ct} - \mu\gamma t - \gamma\mu(w_c + h + k) - \mu\delta(p_b - w_b)}{\delta\mu(\gamma - \delta)\left(1 + \theta_h^b\right) + \lambda\gamma(\mu - \lambda)(1 + \theta_s^s)}\right] \tag{4}$$

$$p_s^t = \frac{\lambda}{\mu} p_j^{ct} - t - \frac{\lambda}{\mu}(\mu - \lambda)\left[\frac{\gamma\lambda P_j^{ct} - \mu\gamma t - \gamma\mu(w_c + h + k) - \mu\delta(p_b - w_b)}{\delta\mu(\gamma - \delta)\left(1 + \theta_h^b\right) + \lambda\gamma(\mu - \lambda)(1 + \theta_s^s)}\right] \tag{5}$$

The post-tax equilibrium cost of soda firms is

$$p_s^{ft} = w_c + \frac{\delta}{\gamma}(p_b - w_b) + h + k + \frac{\delta(\gamma-\delta)}{\gamma}\left[\frac{\gamma\lambda P_j^{ct} - \mu\gamma t - \gamma\mu(w_c + h + k) - \mu\delta(p_b - w_b)}{\delta\mu(\gamma-\delta)(1+\theta_h^b) + \lambda\gamma(\mu-\lambda)(1+\theta_s^s)}\right] \tag{6}$$

The post-tax equilibrium producer prices of HFCS and corn are derived as

$$p_h^t = w_c + \frac{\delta}{\gamma}(p_b - w_b) + h + \frac{\delta(\gamma-\delta)}{\gamma}\left[\frac{\gamma\lambda P_j^{ct} - \mu\gamma t - \gamma\mu(w_c + h + k) - \mu\delta(p_b - w_b)}{\delta\mu(\gamma-\delta)(1+\theta_h^b) + \lambda\gamma(\mu-\lambda)(1+\theta_s^s)}\right] \tag{7}$$

$$p_c^t = w_c + \frac{\delta}{\gamma}(p_b - w_b) + \frac{\delta(\gamma-\delta)}{\gamma}\left[\frac{\gamma\lambda P_j^{ct} - \mu\gamma t - \gamma\mu(w_c + h + k) - \mu\delta(p_b - w_b)}{\delta\mu(\gamma-\delta)(1+\theta_h^b) + \lambda\gamma(\mu-\lambda)(1+\theta_s^s)}\right] \tag{8}$$

The equilibrium conditions in the fruit juice supply chain under the SSB tax are

$$x_j^t = \frac{\varepsilon\left[c(\mu-\lambda) + P_s^{ct} - w_p - m\right] - \upsilon(p_f - w_f)}{\upsilon(\varepsilon-\upsilon)(1+\theta_p^b) + \varepsilon(\mu-\lambda)(1+\theta_j^s)} \tag{9}$$

$$p_j^{ct} = c(\mu-\lambda) + p_s^{ct} - (\mu-\lambda)\left\{\frac{\varepsilon\left[c(\mu-\lambda) + P_s^{ct} - w_p - m\right] - \upsilon(p_f - w_f)}{\upsilon(\varepsilon-\upsilon)(1+\theta_p^b) + \varepsilon(\mu-\lambda)(1+\theta_j^s)}\right\} \tag{10}$$

$$p_j^{ft} = w_p + \frac{\upsilon}{\varepsilon}(p_f - w_f) + m + \frac{\upsilon(\varepsilon-\upsilon)}{\varepsilon}\left\{\frac{\varepsilon\left[c(\mu-\lambda) + P_s^{ct} - w_p - m\right] - \upsilon(p_f - w_f)}{\upsilon(\varepsilon-\upsilon)(1+\theta_p^b) + \varepsilon(\mu-\lambda)(1+\theta_j^s)}\right\} \tag{11}$$

$$p_p^t = w_p + \frac{\upsilon}{\varepsilon}(p_f - w_f) + \frac{\upsilon(\varepsilon-\upsilon)}{\varepsilon}\left\{\frac{\varepsilon\left[c(\mu-\lambda) + P_s^{ct} - w_p - m\right] - \upsilon(p_f - w_f)}{\upsilon(\varepsilon-\upsilon)(1+\theta_p^b) + \varepsilon(\mu-\lambda)(1+\theta_j^s)}\right\} \tag{12}$$

where x_j^t is the equilibrium quantity of fruit juice; and $p_j^{ct}$and p_p^t are the equilibrium consumer price of fruit juice and producer price of processed fruit under the SSB tax, respectively.

Solving the equilibrium expressions for p_j^{ct} and p_s^{ct} simultaneously and substituting into equations (1) to (12), we can derive the equilibrium prices and quantities as functions of the exogeneous parameters of the model such as the tax, consumer preference and producer cost enhancement factors, and firms' market power. This enables us to quantify the impact of the

policy on the welfare of the interest groups involved under different empirically relevant scenarios, which is the focus of the simulation analysis of this paper.

Replication note: Replication of the post-tax equilibrium proceeds as follows: (i) specify the exogenous parameters and the tax level; (ii) substitute the equilibrium quantity of soda given in equation (3) into equations (4)–(8) to obtain post-tax soda prices and costs; and (iii) solve equations (9)–(12) to derive the equilibrium quantity and prices in the fruit juice market. All calculations are deterministic and can be implemented using standard spreadsheet or mathematical software by using relevant parameters in Table 1.
